# Physical activity level and sport participation in association with academic achievement in physical education among adolescents

**DOI:** 10.3389/fspor.2025.1564984

**Published:** 2025-05-09

**Authors:** Ruben Vist Hagen, Monika Haga, Ole Johan Sando, Håvard Lorås

**Affiliations:** ^1^ Department of Teacher Education, Faculty of Social and Educational Sciences, Norwegian University of Science and Technology, Trondheim, Norway; ^2^ Department of Physical Education and Health, Queen Maud University College of Early Childhood Education, Trondheim, Norway

**Keywords:** lower secondary school, youth, grading, leisure-time activities, assessment

## Abstract

**Introduction:**

Physical education (PE) teachers’ assessment practices and frequently applied teaching content suggest that pupils’ academic achievement in PE may be influenced by individual differences in pupil-related factors such as leisure-time physical activity levels and sport participation. Although physical activity could be considered an inherent part of PE, neither of these factors are explicit assessment criteria in the Norwegian PE curriculum. Hence, the current study aimed to investigate the association between leisure-time physical activity levels and sport participation (e.g., team ball, endurance, aesthetic, and strength sports) on academic achievement in PE for pupils attending lower secondary school (13–16 years).

**Methods:**

A total of 169 boys, 174 girls, and 6 who identified as other gender (*N* = 349) participated in the study. The pupils answered an online questionnaire to collect data on their last-received grade in PE, as well as different questionnaire items from the Young-HUNT study related to leisure-time physical activity and sport participation.

**Results:**

The main analysis, via stepwise regression, revealed that a greater frequency of pupils’ leisure time physical activity and participation in endurance type and team/ball sports was significantly associated with a better grade in PE. The pupils who attended PE and similar subjects more frequently were also significant predictors in the regression model. The final mediation analysis indicated that participation in endurance type and team/ball sports mediated the relationship between the frequency of leisure-time physical activity and the grade in PE.

**Discussion:**

The results are discussed in relation to the Norwegian PE curriculum, indicating a misalignment between learning outcomes and teachers’ assessment and grading practice.

## Introduction

The given grade in physical education (PE), as a proxy for academic achievement, should reflect a pupil’s attained level of knowledge and skills in accordance with the national curriculum’s expected learning outcomes (1, 2). Therefore, the grade in PE must reflect a valid assessment procedure, as it functions as a selection instrument in the progress toward higher education and/or employment when added up with grades from the other school subjects (3). As such, it is also essential that the PE grade is an objective measure of academic achievement and that possible influential factors are accounted for and properly integrated into the assessment procedures.

**Figure 1:**
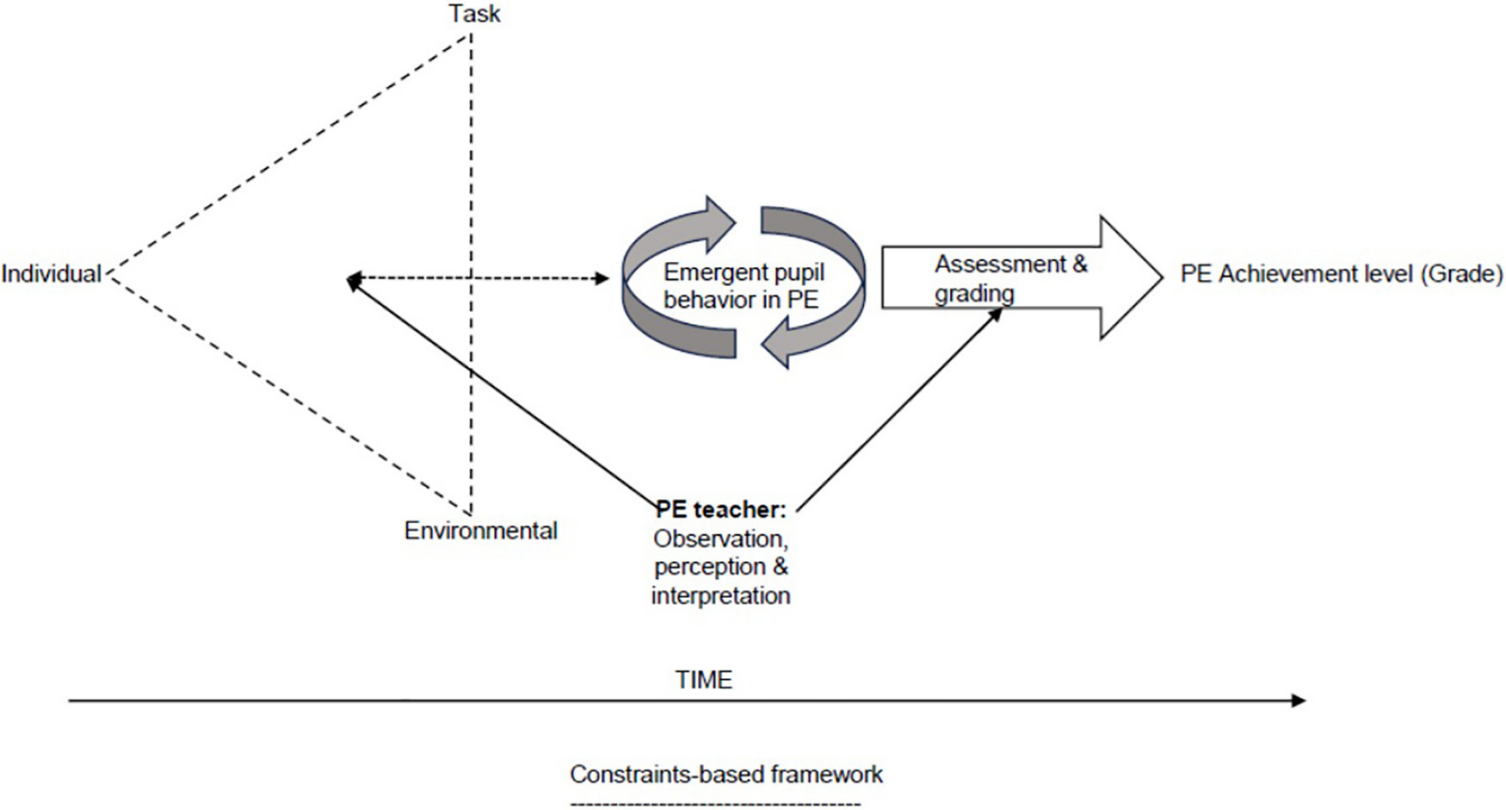
Academic achievement as an outcome of interacting constraints [adapted from Newell (4)] (8).

In the perspective of a constraints-bed framework (4), there are multiple constraints outside of that embodied within the curriculum that influence the evaluation of academic achievement and thus represent a challenge for pupils, teachers, and educators in PE. As PE teachers use observation as the primary means for assessing pupils' academic achievement level (5, 6), understanding how pupil behavior emerges is necessary for the conceptualization of academic achievement in the subject. Under this framework, teachers assess pupil behavior in PE that emerges from the interacting individual, environmental, and task constraints operating on a session-by-session basis (7). Thus, academic achievement in PE could be understood as an outcome of pupil behavior that satisfies the interacting task and environmental constraints that operate within the subject (7, 8) (Figure 1). Through their manipulation of the task and environmental constraints, the curriculum and the teacher can be viewed as key factors in influencing the type of individual constraints that are given value in relation to academic achievement in PE.

Through its stated learning outcomes and accompanying text, the Norwegian PE curriculum describes the goal-directed behavior that is to be learned, informs on the teaching content, and sets the physical environment for learning (1). From the perspective of constraints, it therefore provides the formal environmental and task constraints imposed on the individual pupil from which he or she must accommodate for behavior to be deemed successful (8). The Norwegian PE curriculum’s stated learning outcomes involve the psychomotor, social, cognitive, and affective domains (1, 9). For this study’s particular cohort, learning outcomes are to be achieved by the end of study year 10. In essence, these learning outcomes focus on pupils’ ability to make use of, practice, and develop different skills and knowledge within various movement contexts, to reflect on how different presentations of the body in media and society may affect body image and physical activity, and to acquire the skills and knowledge to practice sustainable and safe use of the nature and its elements (1). Additionally, learning outcomes also state that pupils should develop the ability to cooperate and interact with their peers, acknowledge individual differences, and support their peers’ learning processes (1). Accompanying the curriculum’s learning outcomes, pupils’ efforts should also be part of teachers’ assessment and grading of pupils’ achievement in Norwegian PE. Within the curriculum (1), the effort is operationalized as pupils portray independence in their learning process, continuously keep trying to work on challenges specific to the subject, and challenge their own physical capacities. According to the curriculum, it should be perceived as a positive in terms of assessment and grading that the pupils keep trying even when progress in performance or skill acquisition is lacking (1).

As identified by Newell and Valvano (10), PE teachers represent a key constraint on pupils' emergent behaviors, as they are the ones responsible for shaping both environmental (e.g., outdoor or indoor sessions and socio-environmental factors) and task constraints (e.g., playing games or practicing specific skills) that shape the overall design of sessions. Compared to other school subjects, PE teachers experience a considerable degree of autonomy and are primarily responsible for the assessment and grading of the subject (5, 11, 12). In the perspective of constraints, the teachers thus have a key two-sided role in (1) designing the learning tasks and environment of PE classes that, consequently, impact and shape the student behavior (13) and (2) assessing and grading the emergent pupil behavior according to the national curriculum’s expected learning outcomes and establishing the individual level of academic achievement across multiple sessions.

Individual constraints represent unique structural and functional characteristics of pupils and include factors related to their physical, physiological, cognitive, and emotional build-up (7). These person-related factors, in interaction with other constraints, also contribute to the emergence of pupil behavior in PE and thus potentially impact the behavioral features that teachers value (or not) in the assessment and grading process. Indeed, academic achievement in PE has previously been linked to a variety of individual constraints. For instance, psychological factors such as greater physical self-perceptions (14) and autonomous forms of motivation (15, 16) have previously been associated with higher grades in PE. Furthermore, relative age (e.g., 17, 18), timing of biological maturity (18), and physical fitness levels (19) have additionally been identified as influential factors among Norwegian pupils’ differential academic achievement levels in the subject. In the Norwegian context, such findings have been argued to reflect assessment practices that are not necessarily in confluence with the curriculum’s stated learning outcomes and rather align with the task and environmental constraints strongly influenced by teachers’ perceptions of a more skill- and sport-oriented subject (8).

According to teachers’ assessment practices, it is suggested that pupils need to perform well in sport-related skills and have a certain physical fitness level to achieve high grades in PE (6, 20–22). Therefore, the impact of pupils' involvement in sports outside the school setting and their physical activity level has been acknowledged as influential in determining their potential for achieving higher grades (20, 23). This recognition extends to Norwegian PE teachers who emphasize the importance of pupils demonstrating specific team sport skills (such as ball games) and showing proficiency in physical fitness components to attain the highest grades (22). Indeed, Vedøy et al. (24) report a significant positive association between objectively measured physical activity and academic achievement in PE in a Norwegian adolescent cohort. Notably, this association was more pronounced among girls than boys. Moreover, Leirhaug (3) discovered a positive correlation between pupils' weekly physical activity levels and their grades in PE, while a single item assessing pupils’ participation in competitive sports did not show a statistically significant relationship. Meanwhile, Wiium and Säfvenbom (25) observed a positive association between both self-organized physical activity and participation in organized sports with grades in PE. However, their findings also indicated that pupils with higher grades were more likely to engage in both organized sports and self-organized physical activity, compared with pupils solely involved in organized sports.

The suggested associations between pupils’ grade in PE and their leisure-time physical activity and sport participation may be founded in two arguments. Firstly, physical activity serves as a key determinant of individual physical fitness (26). Consequently, it is plausible to suggest that the levels and intensity of leisure-time physical activity may indirectly impact pupils' achievement in PE by influencing their physical fitness levels. Furthermore, Norwegian PE teachers interpret the assessment and grading criterion of effort as the capacity to visibly exhibit strenuous physical work, as outlined by Aasland and Engelsrud (27). Consequently, pupils with higher physical fitness levels may possess the ability to exert greater effort and, through the activities chosen by teachers, demonstrate higher levels of speed, endurance, and strength compared with their counterparts with lower physical fitness levels. Secondly, as quantity and quality of practice are argued as robust explanatory mechanisms of learning and performance (28–30), pupils’ academic achievement level could be influenced by the movement experience they acquire during leisure-time physical activity. The subject of PE seems to be dominated by a sport-oriented teaching content (31). More specifically, Norwegian PE teachers seem to heavily rely on fitness training and team/ball sports as their choice of teaching activities (32, 33). Accompanied by the abovementioned assessment and grading practices, individual pupils’ amount of experience with certain sports may therefore provide a carryover effect in accordance with these teacher-influenced task constraints operating in the subject.

### Aim of the current study

Based on the presented considerations, which highlighted that pupil's leisure-time sport experiences and physical activity levels may potentially be individual constraints that influence academic achievement through mediating observed (and graded) behaviors in PE, the principle aim of the current study was to investigate the association between leisure-time physical activity levels and sport participation (e.g., team/ball, endurance, aesthetic, and strength sports) on academic achievement in PE for pupils attending lower secondary school (13–16 years). It was hypothesized that (1) pupils with higher physical activity levels would receive higher grades in PE compared with those with lower physical activity levels and (2) pupils who participate more frequently in specific sports, especially team/ball games or sports that require components of physical fitness, receive higher grades in PE compared with peers who participate less frequently.

## Materials and methods

### Participants

A convenience sample of 349 pupils was recruited from two public Norwegian lower secondary schools (study years 8–10) to answer an online questionnaire. A total of 143 and 206 pupils represented study years 9 and 10, respectively. Aiming to include pupils of different sociocultural and socio-environmental backgrounds, one of the participating schools was located in a rural community, and the other was located in an urban setting in a larger Norwegian city. All public schools within the Norwegian education system are regulated by and follow the same curricula. They are also coeducational. To be included in the study, all participating pupils had to previously receive a grade in PE and follow a normal school progression in line with their date of birth.

### Procedure

Data collection was conducted during a 2-month period from December 2021 to January 2022. By using their personal computer, participating pupils answered the questionnaire via the online service Nettskjema (UIO) while seated at their desks during normal school hours. Trained practitioners were present in the initial parts of the data collection to answer any questions regarding the wording or format of the questions or to address any potential technical issues with the online questionnaire. The pupils were encouraged to answer questions as truthfully as possible. They were also informed that participation would not influence their grade in the subject and that their teachers would not be allowed to see their answers. The questionnaire included items concerning their gender, age, year of study, body weight, and height and items concerning their leisure-time physical activity and sport participation. Additionally, the questionnaire asked pupils to self-report the grade they last received in PE. Given the time of data collection, this would be the grade they received at the end of their previous school year. In conjunction with the grade, the items for leisure-time physical activity and sport participation are of main interest in this study and therefore addressed in more detail in the following section.

Prior to the data collection, pupils, and their legal guardians, were informed of the purpose of the study, that participation was voluntary, that they could withdraw from the study at any time, and that all data were collected and treated anonymously. The study was approved by the Norwegian Agency for Shared Services in Education and Research (SIKT) (previously known as the Norwegian Centre for Research Data) (project ID: 169464). After a consultation between SIKT and the first author of this study, it was deemed adequate that the pupils themselves consented to participate by answering and completing the online questionnaire. This was made possible due to the nature of the involved questions and the use of Nettskjema as the online service ensuring anonymity and concealment of IP addresses.

### Measurements

#### Academic achievement in physical education

As an indicator of academic achievement in PE, pupils reported their last-received grade in PE at the time of data collection. It is important to note that although self-reported grades are considered relatively accurate representations of the actual teacher-reported achievement levels, they may be subject to some overestimation of achievement on pupils’ part (34). In the Norwegian education system, grades are not used as part of teachers’ end-of-term or end-of-year assessment and grading until pupils attend year 8 in lower secondary school, which is the first of three years at that level. The grading scale ranges from 1 to 6, where a grade of 1 is described as “very low competence” and a grade of 6 as “excellent competence” for all school subjects (35).

#### Leisure-time physical activity level

The current study made use of relevant questions from the Trøndelag Health Study (Young-HUNT 4) to assess pupils’ leisure-time physical activity and sport participation. The Young-HUNT study is aimed at Norwegian 13–19-year-olds and, in the period between 2017 and 2019, captured data from 8,066 adolescents concerning, among other aspects, questions related to their physical activity behaviors (36).

The single items in the questionnaire address different aspects of physical activity behavior, namely, frequency, duration, and/or intensity. Two items address the extent to which pupils are physically active during their leisure time: “Outside of school: how often do you participate in sports or physical activity so much that you get out of breath and/or sweat?” (frequency), and “Outside of school: how many hours a week do you participate in sports or physical activity so much that you get out of breath and/or sweat?” (duration/time). The wording “get out of breath and/or sweat” indicates moderate-to-vigorous intensity of the physical activity behavior (37). The frequency-related item ranges between *never* (1) to *every day* (6), and the duration item ranges from *none* (1) to *7 h a day* (6), on a six-point Likert scale. These single items are translated from the World Health Organization in Schoolchildren (HBSC) questionnaire. They have previously been validated in a Norwegian adolescent sample (38).

An additional three items were included addressing the frequency of participating in organized, “How often do you participate in organized training (with sport teams or associations)?”; unorganized, “How often do you participate in unorganized training with others (e.g., playing football with friends, and training at the gym/group classes)?”; and training on own initiative, “How often do you train individually (training alone, on own initiative)?”. All items involved participants evaluating the frequency ranging from *never* (1) to *four times a week or more* (5). The first two items have more recently been applied to a Norwegian adolescent sample (36).

#### Duration of participation in PE

Another six-point Likert scale item, ranging from *none* (1) to *5 h or more* (6) from the Young-HUNT study concerning duration in PE was included: “How many school hours a week do you participate in Physical education at school?”. Of note, this item also, in brackets, highlights for the participant that it involves other optional subjects in school of a physical activity-related nature. At the lower secondary school level, this would, in addition to PE, include the optional subject of physical activity and health. This subject is concerned with facilitating pupils’ understanding, interest, and development of a health-promoting lifestyle via physical activity (39).

#### Leisure-time sport participation

Finally, a single item from the Young-HUNT study was used to more specifically address the frequency in which pupils were involved with different sports in their leisure time. The item is worded, “How often do you participate in the following activities?”, with potential answers ranging from *never* (1) to *four times a week or more* (5). The available sport categories are endurance sports (e.g., running, cross-country skiing, cycling, swimming, track, and field), team/ball sports (e.g., football, volleyball, handball, and ice hockey), aesthetic sports (e.g., dance, gymnastics, and aerobics), martial arts/strength sports (e.g., judo, karate, taekwondo, boxing, and powerlifting), resistance training (e.g., bodybuilding and fitness training), technical sports (e.g., horse riding, track and field, jumping, and skateboarding), ski sports (e.g., alpine skiing, snowboarding, and telemark), outdoor (“friluftsliv”) (e.g., hiking and skiing trip), and training at the gym. This item has also previously been used in a Norwegian adolescent sample (37).

### Statistical analysis

Prior to the main analysis, missing data (2.25%) were assessed and deemed missing completely at random (40). Missing data were treated by the multiple imputation method (41) which resulted in five iterations. The average score of these five iterations was used to replace the missing values for each case. The distribution of normality was also assessed by investigating the histograms and Q–Q plots of the variables analyzed.

The pupils' responses to the questionnaire were imported into STATA MP version 18 (StataCorp) for statistical analyses. Descriptive statistics, correlation analysis, regression analysis, and mediation analysis were performed to answer the research questions in this paper. Descriptive analysis provided mean, standard deviation, and range (minimum and maximum values) for all variables used in the study. Regression analysis (42) was employed with pupils' grades as the dependent variable. This analysis aimed to examine the association between grade and background variables such as age, gender, and BMI. Next, each of the variables describing leisure-time physical activity levels and leisure-time sport participation was added to separate models, to test their relationship to PE grade while controlling for age, gender, and BMI. Correlation analysis was subsequently conducted with variables showing significant associations with PE grades in the initial regression analysis, to explore relationships among these variables.

Stepwise regression analysis (42) was utilized to identify the most influential variables related to leisure-time physical activity levels and sport participation on PE grades. This process involved sequentially adding variables, beginning with background factors in Step 1, followed by leisure-time physical activity variables in Step 2, and leisure-time sport participation variables in Step 3. Only variables significantly associated with PE grades progressed to the next step, resulting in the final regression model (Step 4).

Finally, mediation analysis was performed to examine the extent to which leisure-time sport participation mediated the relationship between leisure-time physical activity levels and PE grades. The mediation analysis was conducted using the medsem package (43), employing structural equation modeling (SEM) with an adjusted Baron and Kenny approach (44) to assess mediation effects.

## Results

### Descriptive statistics

The average age of the participating pupils (*N* = 349) was 14.6 years old (SD = 0.6). Among the pupils, 169 identified as males, 174 as females, and 6 other genders. The six in the other category are not included in the analysis where gender is included. The participant's average BMI was 20.7 (SD = 2.8), and the mean grade in PE was 4.61 (SD = 0.68) for the boys and 4.40 (SD = 0.74) for the girls. These average scores are slightly below the national average of 2021 for 10th grade (boys, 4.8; girls, 4.7) (45). Table 1 presents the descriptive statistics on age, BMI, and grade and the questions used to measure the pupils' physical activity levels and sport participation.

**Table 1:** Mean, standard deviation (SD), minimum, and maximum values for the variables used in the study (*n* = 349).

Variable	Mean	SD	Min	Max
Pupil characteristics				
Age	14.6	0.6	13	16
BMI	20.7	2.8	14	31
Grade	4.5	0.7	2	6
Leisure-time PA levels				
PA frequency	4.4	1.2	1	6
PA hours	4.4	1.5	1	6
PE participation	3.4	1.0	1	5
Organized activity	3.2	1.6	1	5
Non-organized activity w/others	2.7	1.3	1	5
Training own initiative	3.1	1.3	1	5
Leisure-time sport participation				
Endurance sports	2.9	1.2	1	5
Team ball sports	2.9	1.7	1	5
Aesthetic sports	1.5	1.0	1	5
Combat strength	1.5	1.0	1	5
Strength sports	2.9	1.3	1	5
Technical sports	1.4	0.9	1	5
Ski sports	1.7	0.9	1	5
Outdoor life	2.3	1.1	1	5
Fitness center	2.2	1.4	1	5

PA, physical activity; PE, physical education.

To explore the impact of age, gender, and BMI on grade, a regression analysis was conducted with grade as the dependent variable. Age, gender, and BMI were added to the model as independent variables. While controlling for the effect of the other independent variables in the model, the pupils' age was positively associated with grade in PE (B = 0.15, *p* = 0.027). Boys' grades were significantly higher than girls (B = 0.20, *p* = 0.009), and pupils with higher BMI received a significantly lower grade (B = −0.03, *p* = 0.037). This first pupil characteristics model explained a limited amount of the variance in grade (*R*² = 0.04) and predicted that a female 13-year-old pupil with a BMI of 27 receives a grade in PE of 3.8, compared with a grade in PE of 4.6 for a 15-year-old boy with a BMI of 22.

### Initial regression analysis: background factors and physical activity levels

Next, each of the variables describing the physical activity level and characteristics were added to this model separately to identify which variables were associated with the grade in PE. Controlling for the pupils' age, gender, and BMI, the response to the variable's physical activity frequency (B = 0.23, *p* < 0.001), physical activity hours (B = 0.18, *p* < 0.001), PE participation (B = 0.23, *p* < 0.001), organized activity (B = 0.15, *p* < 0.001), and training on own initiative (B = 0.12, *p* < 0.001) was positively associated with PE grade. The variables describing non-organized activity were statistically unrelated to grade in PE. For the variables describing the characteristics of the pupils' physical activity while controlling for age, gender, and BMI, participation in endurance sports positively related to PE grade (B = 0.14, *p* < 0.001). Similar associations were found for team/ball sports (B = 0.15, *p* < 0.001), strength sports (B = 0.12, *p* < 0.001), ski sports (B = 0.11, *p* = 0.010), and outdoor life (B = 0.10, *p* = 0.005). The variables describing participation in aesthetic sports, combat strength sports, technical sports, and fitness center were statistically unrelated to PE grades.

### Correlation analysis

Since these variables are all related to the pupils' physical activity levels and characteristics, we can expect them to be correlated. A correlation analysis included the variables significantly associated with grade to explore these associations. This analysis demonstrates that physical activity frequency and activity hours are highly correlated (*r* = 0.74), and these two variables also show a similar correlation to the other variables in the analysis. Moreover, team/ball sports correlate highly to organized activity (*r* = 0.71). The complete results from the correlation analysis are found in Table 2.

**Table 2:** Correlation analysis for the variables used to measure pupils' physical activity levels and characteristics with a significant impact on PE grade (*n* = 349).

Variable	1	2	3	4	5	6	7	8	9	10
1. PA frequency	−									
2. PA hours	0.74***	−								
3. PE participation	0.29***	0.27***	−							
4. Organized activity	0.57***	0.62***	0.21***	−						
5. Training own initiative	0.43***	0.39***	0.15**	0.01	−					
6. Endurance sports	0.37***	0.35***	0.12*	0.27***	0.30***	−				
7. Team ball sports	0.49***	0.52***	0.28***	0.71***	0.01	0.15*	−			
8. Strength sports	0.41***	0.41***	0.20***	0.11*	0.62***	0.21***	0.14**	−		
9. Ski sports	0.10	0.14*	0.12*	0.08	0.17**	0.20***	0.08	0.12*	−	
10. Outdoor life	0.08	0.00	0.14**	−0.02	0.18**	0.24***	0.01	0.08	0.29***	−

PA, physical activity; PE, physical education.

* *p* < 0.05.

** *p* < 0.01.

*** *p* < 0.001.

### Stepwise regression analysis: identifying key predictors of PE grade

To further test what physical activity characteristics most greatly influenced grade in PE, a stepwise regression analysis (Table 3) was performed. Following the high amount of shared variance between physical activity frequency and physical activity hours, as well as between team/ball sports and organized activity, was only one from each of these pairs of variables included in the further analysis. Full models for all variables are available upon request from the corresponding author. Physical activity frequency was chosen over physical activity hours since this variable demonstrated the most substantial relationship with PE grade when the variables were tested individually. Team/ball sports were selected over organized activity since team/ball sports provide more information as to what activity the pupil is engaged in compared with the more overarching variable describing different types of organized sports.

**Table 3:** Stepwise regression analysis for pupils' PE grade (*n* = 343).

Variable/Model	Model 1	Model 2	Model 3	Model 4
	Coeff. (std.err)	Coeff. (std.err)	Coeff. (std.err)	Coeff. (std.err)
Constant	2.7 (1.0)**	2.0 (0.9)*	1.8 (0.9)*	1.8 (0.9)*
Pupil characteristics				
Age	0.16 (0.07)*	0.11 (0.06)*	0.12 (0.6)	0.13 (0.06)*
Boy	0.20 (0.08)**	0.07 (0.07)	0.06 (0.07)	0.06 (0.07)
BMI	−0.03 (0.01)*	−0.03 (0.01)*	−0.03 (0.01)*	−0.03 (0.01)**
Physical activity levels				
PA frequency		0.18 (0.03)***	0.09 (0.04)*	0.11 (0.04)**
PE participation		0.16 (0.04)***	0.12 (0.04)**	0.14 (0.04)***
Training own initiative		0.04 (0.03)		
Sport participation				
Endurance sports			0.06 (0.03)*	0.08 (0.03)**
Team ball sports			0.09 (0.02)***	0.09 (0.02)***
Strength sports			0.04 (0.03)	
Ski sports			0.04 (0.04)	
Outdoor life			0.04 (0.03)	
Model statistics				
*F*-statistics	5.1**	16.6***	13.0***	17.5***
*R* ^2^	0.04	0.23	0.28	0.27
Adj. *R*^2^	0.04	0.21	0.26	0.25
AIC	737	669	653	652
BIC	752	696	694	684

PA, physical activity; PE, physical education.

* *p* < 0.05.

** *p* < 0.01.

*** *p* < 0.000.

The first step of the regression analysis (Model 1) involved running a model with the pupil characteristics age, boy, and BMI. In the second step (Model 2), the variables describing physical activity frequency, PE participation, and training on one's own initiative were included. Physical activity frequency and PE participation were positively and significantly related to PE grade when controlling for these other variables describing physical activity levels, while training on one's own initiative was not statistically associated with PE grade in this model. Therefore, this variable was not included in the third step of the analysis, where the variables describing physical activity characteristics were included. The level of participation in endurance sports and team/ball sports was positively and significantly related to PE grade. Strength sports, ski sports, and outdoor life were statistically unrelated to PE grade in this model and were excluded in Model 4. The final model suggests that physical activity frequency (B = 0.11, *p* = 0.002), PE participation (B = 0.14, *p* < 0.001), endurance sports (B = 0.08, *p* = 0.009), and team/ball sports (B = 0.09, *p* < 0.001) are positively associated with PE grade also in a multiple regression model where we include several measures on the pupils' physical activity levels and characteristics.

Predictions based on Model 4 indicate that a sedentary (1 on physical activity frequency) 15-year-old boy with a BMI of 22 who rarely participates in PE (1 on PE participation) and who is not engaged in endurance sports or team/ball sports (1 on both variables) would receive a PE grade of 3.5. A highly active boy (6 on physical activity frequency) who scores max on PE participation (5) of the same age and with the same BMI as the sedentary boy who is not engaged in endurance sports or team/ball sports (1 on both variables) would be estimated with a grade of 4.6. If the same boy were somewhat involved in endurance sports (3 on this variable) and highly engaged in team/ball sports (5 on this variable), the grade would be predicted to be 5.1. These results suggest that both physical activity levels and characteristics influence PE grade to a substantial extent.

### Mediation analysis

Furthermore, since endurance sports and team/ball sports are a part of many pupils' weekly physical activity, these activities may mediate the positive relationship between physical activity frequency and grade. To explore this possible relationship, two mediation analyses were conducted. The mediation analysis revealed evidence of partial mediation for both endurance sports and team/ball sports, suggesting that the mediation effect of these sports partially explains the relationship between physical activity frequency and academic achievement. The indirect effect of physical activity frequency on grade, mediated by participation in endurance sports, was estimated to be 0.057 (standardized effect). The *z*-value for the indirect effect in the endurance sports mediation model was 2.772, with a corresponding *p*-value of 0.006, indicating that the indirect effect is statistically significant. We calculated the ratio of indirect to total Effect (RIT) for endurance sports as the ratio of the indirect effect to the total effect, resulting in a value of 0.139. This suggests that approximately 14% of the total effect of physical activity frequency on PE grade is mediated by participation in endurance sports. The ratio of indirect to direct effect (RID) for endurance sports, calculated as the ratio of the indirect effect to the direct effect, is approximately 0.161. This indicates that the mediated effect, through participation in endurance sports, is approximately 0.2 times as large as the direct effect of physical activity frequency on academic achievement.

The indirect effect of physical activity frequency on PE grade, mediated by participation in team/ball sports, was estimated to be higher than for endurance sports, with a standardized effect of 0.102. The *z*-value for the indirect effect was 3.685, with a corresponding *p*-value of <0.001, indicating that the indirect effect is statistically significant. We calculated the RIT as the ratio of the indirect effect to the total effect, resulting in a value of 0.246. This indicates that participation in team/ball sports mediates approximately 25% of the total effect of physical activity frequency on PE grade. The RID, calculated as the ratio of indirect and direct effects, is approximately 0.326. This suggests that the mediated effect, through participation in team-/ball sports, is approximately 0.3 times as large as the direct effect of physical activity frequency on PE grade.

These findings from the mediation analysis collectively indicate that participation in endurance sports and team/ball sports significantly mediates the association between physical activity frequency and PE grade. It is important to note that these mediation effects are partial, suggesting that while these sports explain a significant portion of the relationship, other factors may also contribute to the observed relationship between physical activity frequency and PE grade.

## Discussion

The current study’s main aim was to investigate the association between pupils’ academic achievement in PE and their leisure-time physical activity and sport participation in a Norwegian sample at lower secondary school. It was hypothesized that pupils with higher leisure-time physical activity levels and sport participation, especially participation in team/ball games and sports that require physical components, would receive higher grades as opposed to peers with lower leisure-time physical activity levels and sport participation. The main findings indicated that the frequency in which pupils conducted physical activity in their leisure time and participated in endurance type and team/ball sports were significantly associated with the grade pupils received in PE. Additionally, their frequency of participating in PE and other similar subjects was significantly associated with the grade as well. However, the directionality of this relationship remains unclear. Furthermore, a mediating effect of leisure-time participation in endurance- and team/ball sports on the relationship between the frequency of leisure-time physical activity and pupils’ grade was identified. Participation in endurance sports and team/ball sports mediated approximately 14% and 25% of the frequency of leisure-time physical activity’s total effect on the grade in PE, respectively.

### Physical activity level and academic achievement in physical education

As expected, pupils who reported being more often physically active at a moderate-to-vigorous intensity level received a higher grade in the subject, suggesting a potential association between physical activity levels and academic achievement in PE. As such, the current study’s results provide evidence to support our first hypothesis, while also lending support to previous studies indicating that leisure-time physical activity behavior acts as an individual constraint on academic achievement in PE (3, 24, 25). As an immediate thought, this might be a reasonable finding in that PE is primarily a movement-oriented subject in which learning and development take place via different physical activities as described in the Norwegian PE curriculum. As such, physical activity is an inherent component of the subject (3, 46). However, being physically active, per se, or having specific levels of acquired physical activity during pupils’ leisure time are not criteria of the PE curriculum. Despite this, traditional assessment and grading practices seem to, in part, place value on pupils’ (physical) activity levels in the subject (47). For instance, Leirhaug (3) has previously highlighted that pupils more active during PE lessons received a higher grade in the subject.

As physical activity is considered an influential factor in the development of physical fitness (26), individual differences in leisure-time physical activity could also have an indirect influence on achievement in PE by potentially mediating the relationship between pupils’ grade and physical fitness. The impact of physical fitness has previously been highlighted among Norwegian adolescents in which pupils who portrayed greater levels of endurance and speed, lower body power/strength, and in general greater physical fitness had better grades compared with peers scoring lower on these physical capacities (19). A rationale underpinning these findings may be related to the demands placed upon pupils’ ability to perform by the dominant teaching content of physical exercise activities and team and ball sports (33, 33). More specifically, the enduring use of physical fitness testing evident through both Norwegian (6, 12, 32) and international literature (21, 48, 49) might suggest that, logically, physical fitness, accompanied by a potential mediating effect of physical activity, is partly influential on pupils’ achievement level in the subject. Moreover, it has previously been argued that specific learning outcomes of the Norwegian PE curriculum may themselves place demands in which pupils of greater physical fitness are better equipped to accommodate, work efficiently, and achieve, to a higher degree, the expected learning outcomes to be reached as opposed to their peers of lower physical fitness levels (50). For instance, to attain the learning outcome “exercise and develop skills in a variety of movement activities” (1 p8), better physically fit pupils may have an advantage in their capacity to efficiently practice skills of a particular movement activity.

### Sport participation and academic achievement in physical education

Another main finding of the current study revealed that Norwegian pupils who more frequently participated in endurance and team/ball sports, specifically, during their leisure time were more advantageous in terms of higher grades. While similar to and supporting a previous study on Norwegian adolescents (25), in which involvement in organized sports seemed beneficial for better grades, these findings may offer a more nuanced perspective in highlighting the distinctive influence of specific sports on pupils’ academic achievement in PE. It should be noted that while Leirhaug (3) found a significant statistical association between leisure-time physical activity and the grade in PE, pupils’ participation in, what was referred to as, competitive sports was not. As previously noted, Norwegian PE teachers experience considerable autonomy in their teaching, assessment, and grading practice. Consequently, there is a potential for meaningful variations in teaching and assessment/grading practices between both teachers and schools which ultimately is reflected in pupils’ grades in PE.

Through the initial regression analysis of this study, the frequency of leisure-time participation in endurance, team/ball, strength, and ski sports, as well as outdoor activities, was all positively associated with the grade in PE. Meanwhile aesthetic, combat, and technically oriented sports and training at fitness centers were not statistically associated with the grade. The explanation for why participation in these latter sports was not related to achievement in PE in the current study is probably due to the broadly defined content area of the Norwegian PE curriculum, which provides Norwegian PE teachers with considerable autonomy in their teaching and selection of activities. Although all the assessed sports/activities in this study could arguably be applied in PE to address pupils’ work on the Norwegian curriculum’s learning outcomes (1), the few sports that were significantly associated with the grade indicate that some teaching content is prioritized above others. This assumption is strengthened by the stepwise regression analysis in which only the frequency of participation in the endurance- and team/ball sports acted as significant predictors of pupils’ grade in contrast to strength training, ski sports, or outdoor life, which were included in the same model. These results indicate as to which physical activity characteristics are of importance to academic achievement in PE and, as such, suggest a potential carryover effect in PE. As argued in the introduction, quantity and quality of training (or experience) with particular movement activities are important indicators of performance (28–30). It could therefore be argued that pupils participating more frequently in the significantly associated sports are beneficial due to the reason that these are activities dominating what is taught in PE (31–33). If teachers, additionally, view achievement in PE through the lens of sports (6, 20–23), pupils may potentially be rewarded for their technical and tactical proficiency exceeding that of their less experienced peers. At the same time, the lack of any statistical association between the grade with the other included activities is indicative of the less prioritized content area of the curriculum such as dance, outdoor activities, and swimming (32, 33).

### Methodological considerations and further study

In the following, methodological limitations and strengths are acknowledged to inform potential further studies addressing similar avenues of research. Firstly, it is important to highlight that the current study can make no assumption about causality based on its cross-sectional design. Additionally, the temporal alignment between the reported PE grade and current leisure-time physical activity levels should be considered a limitation, as the study does not account for changes in behavior over time. Future research employing longitudinal designs could better capture the directionality of this relationship. The decentralized education system of Norway, and the associated autonomy experienced by Norwegian PE teachers, suggests that there might be differences in both teaching and assessment/grading practices locally (between teachers) and geographically (between schools), potentially resulting in different factors influencing pupils’ achievement in PE. Meanwhile, tendencies offered by both Norwegian and international literature suggest that a sport-oriented perspective on achievement in PE is present across contexts, which may warrant similarities in what constitutes achievement. Secondly, though a convenience sample may induce selection bias, the current sample’s average grade was similar to, but slightly below, that of the average national achievement level for 10th grade pupils in 2021, which indicates some resemblance of the current sample to that of the general population in Norwegian lower secondary schools. Thirdly, as depicted by the constraints-based framework, pupils’ academic achievement in PE could be understood as the confluence of interacting constraints residing in the individual, task, and environment. In this study, the rationale underpinning the choice to investigate leisure-time physical activity characteristics resides in current knowledge on teaching content, assessment/grading practices, and associated discourse in the subject which can be identified as either task and/or environmental constraints. These constraints are not addressed in this study but, according to the framework, are influential on which individual constraints are of relevance to investigate in association with pupils’ academic achievement in PE.

There is a need for more in-depth studies on this topic, and future research would benefit from adopting a mixed-methods approach. By incorporating qualitative methods, researchers could explore how students experience the value of physical activity during leisure time and its impact on cognitive processes, such as motivation. Additionally, it could examine how students perceive and reflect on the key aspects of the grading process. Furthermore, physical education teachers could be asked to evaluate their grading practices through qualitative interviews.

### Practical application

The observed associations between the grade in PE and pupils' leisure-time physical activities and participation in endurance- and team-/ball sports raise questions about potential biases in teachers' teaching, assessment, and grading practices. While these results do not establish causation, they highlight the need for further investigation into whether certain types of sport participation influence grading. Particularly as none of these individual constraints are depicted as assessment and grading criteria in the Norwegian PE curriculum. Hence, these findings might encourage Norwegian PE teachers to apply a greater variety in the teaching content and be aware of how their perception of what constitutes relevant knowledge and skills in PE may influence pupils’ potential for higher levels of academic achievement in the subject. As there might be considerable individual differences within a teacher’s cohort of pupils, the use and consideration of the constraints-based framework may help teachers approach the subject with a more conscious consideration of pupils’ individual prerequisites/conditions and provide more equal opportunities for learning and achievement in PE.

## Conclusion

The study’s results indicate that there is an association between pupils’ academic achievement in PE and their leisure-time physical activity levels and sport participation, which in large accepts the proposed hypotheses of the study. That frequency of participation in endurance and team/ball sports influences the grade level in PE is perhaps of particular interest. It lends support to previous literature suggesting that teachers may perceive skill and knowledge in a sport-oriented sense, which contrasts the Norwegian PE curriculum’s intentions. Collectively, the study’s results offer additional, and perhaps more nuanced, insight into the influential role of leisure-time physical activity behaviors as individual constraints on academic achievement in PE. More specifically, PE teachers should acknowledge inter-individual differences in leisure-time physical activity and sport participation in their teaching, assessment, and grading practice.

## Data Availability

The original contributions presented in the study are included in the article/Supplementary Material, further inquiries can be directed to the corresponding author.

## References

[1] Norwegian Ministry of Education and Research. Curriculum in physical education (KRO01-05). Available at: https://www.udir.no/lk20/kro01-05?lang=eng (Accessed May 5, 2024).

[2] Regulations Pursuant to the Education Act. §3-3 Assessment in Subject (2006). Available at: https://lovdata.no/forskrift/2006-06-23-724 (Accessed May 5, 2024).

[3] Leirhaug PE. Exploring the relationship between student grades and assessment for learning in Norwegian physical education. Eur Phys Educ Rev. (2016) 22(3):298–314. 10.1177/1356336X15606473

[4] Newell KM. Constraints on the development of coordination. In: Wade MG Whiting HTA, editors. Motor Development in Children: Aspects of Coordination and Control. Dordrecht: Martinus Nijhoff (1986). p. 341–60.

[5] Borghouts LB Slingerland M Haerens L. Assessment quality and practices in secondary pe in the Netherlands. Phys Educ Sport Pedagogy. (2017) 22(5):473–89. 10.1080/17408989.2016.1241226

[6] Prøitz TS Borgen JS. Rettferdig Standpunktvurdering—det (u)muliges Kunst? [Fair Grading—the (im)Possible art?]. Oslo: NIFU STEP (2010). Report 16/2010.

[7] Renshaw I Chow JY Davids K Hammond J. A constraints-led perspective to understanding skill acquisition and game play: a basis for integration of motor learning theory and physical education praxis? Phys Educ Sport Pedagogy. (2010) 15(2):117–37. 10.1080/17408980902791586

[8] Hagen RV. Associations between pupil-related factors and achievement in physical education (PhD thesis). Norwegian University of Science and Technology, Trondheim (2024).

[9] Brattenborg S Engebretsen B. Innføring i Kroppsøvingsdidaktikk [Introduction to Physical Education Didactics]. 4th ed. Oslo: Cappelen Damm Akademisk (2021).

[10] Newell KM Valvano J. Movement science: therapeutic intervention as a constraint in learning and relearning movement skills. Scand J Occup Ther. (1998) 5(2):51–7. 10.3109/11038129809035730

[11] DinanThompson M Penney D. Assessment literacy in primary physical education. Eur Phys Educ Rev. (2015) 21(4):485–503. 10.1177/1356336X15584087

[12] Leirhaug PE MacPhail A Annerstedt C. ‘The grade alone provides no learning’: investigating assessment literacy among Norwegian physical education teachers. Asia Pac J Health Sport Phys Educ. (2016) 7(1):21–36.

[13] Chow JY Davids K Hristovski R Araújo D Passos P. Nonlinear pedagogy: learning design for self-organizing neurobiological systems. New Ideas Psychol. (2011) 29(2):189–200. 10.1016/j.newideapsych.2010.10.001

[14] Vist Hagen R Lorås H Sigmundsson H Haga M. The association between pupil-related psychological factors and academic achievement in physical education. J Teach Phys Educ. (2022) 41(3):532–43. 10.1123/jtpe.2021-0063

[15] Barkoukis V Taylor I Chanal J Ntoumanis N. The relation between student motivation and student grades in physical education: a 3-year investigation. Scand J Med Sci Sports. (2014) 24(5):e406–14. 10.1111/sms.12174 24433528

[16] Cid L Pires A Borrego C Duarte-Mendes P Teixeira DS Moutão JM Motivational determinants of physical education grades and the intention to practice sport in the future. PLoS One. (2019) 14(5):e0217218. 10.1371/journal.pone.0217218 31120973 PMC6592572

[17] Aune TK Pedersen AV Ingvaldsen RP Dalen T. Relative age effect and gender differences in physical education attainment in Norwegian school children. Scand J Educ Res. (2017) 61(3):369–75. 10.1080/00313831.2016.1148073

[18] Vist Hagen R Haga M Sigmundsson H Lorås H. The association between academic achievement in physical education and timing of biological maturity in adolescents. PLoS One. (2022) 17(3):e0265718. 10.1371/journal.pone.0265718 35303041 PMC8932553

[19] Vist Hagen R Lorås H Sigmundsson H Haga M. Association between motor competence, physical fitness, and academic achievement in physical education in 13-to 16-year-old school children. Front Sports Act Living. (2022) 3:774669. 10.3389/fspor.2021.774669 35128395 PMC8810521

[20] Hay PJ Macdonald D. Evidence for the social construction of ability in physical education. Sport Educ Soc. (2010) 15(1):1–18. 10.1080/13573320903217075

[21] Redelius K Fagrell B Larsson H. Symbolic capital in physical education and health: to be, to do or to know? That is the gendered question. Sport Educ Soc. (2009) 14(2):245–60. 10.1080/13573320902809195

[22] Aasland E Walseth K Engelsrud G. The constitution of the ‘able’ and ‘less able’ student in physical education in Norway. Sport Educ Soc. (2020) 25(5):479–92. 10.1080/13573322.2019.1622521

[23] Svennberg L. Swedish Pe teachers’ understandings of legitimate movement in a criterion-referenced grading system. Phys Educ Sport Pedagogy. (2017) 22(3):257–69. 10.1080/17408989.2016.1176132

[24] Vedøy IB Anderssen SA Tjomsland HE Skulberg KR Thurston M. Physical activity, mental health and academic achievement: a cross-sectional study of Norwegian adolescents. Ment Health Phys Act. (2020) 18:100322. 10.1016/j.mhpa.2020.100322

[25] Wiium N Säfvenbom R. Participation in organized sports and self-organized physical activity: associations with developmental factors. Int J Environ Res Public Health. (2019) 16(4):1–16. 10.3390/ijerph16040585 PMC6406465 30781609

[26] Blair SN Cheng Y Holder JS. Is physical activity or physical fitness more important in defining health benefits? Med Sci Sports Exerc. (2001) 33(6):S379–99. 10.1097/00005768-200106001-00007 11427763

[27] Aasland E Engelsrud G. “Det er lett å se hvem av dere som har god innsats”. Om elevers innsats og lærerens blikk i kroppsøving [“it’s easy to see who of you that have good effort”. About pupils’ effort and the teacher’s view in PE]. J Res Arts Sports Educ. (2017) 1:5–17. 10.23865/jased.v1.889

[28] Baker J Horton S. A review of primary and secondary influences on sport expertise. High Abil Stud. (2004) 15(2):211–28. 10.1080/1359813042000314781

[29] Baker J Young B. 20 Years later: deliberate practice and the development of expertise in sport. Int rev Sport Exerc Psychol. (2014) 7(1):135–57. 10.1080/1750984X.2014.896024

[30] Davids K Baker J. Genes, environment and sport performance: why the nature-nurture dualism is no longer relevant. Sports Med. (2007) 37(11):961–80. 10.2165/00007256-200737110-00004 17953467

[31] Kirk D. Physical Education Futures. London: Routledge (2010). p. 184.

[32] Moen KM Westlie K Bjørke L Brattli VH. Når Ambisjon Møter Tradisjon: En Nasjonal Kartleggingsstudie av Kroppsøvingsfaget i Grunnskolen (5.–10. Trinn) [When Ambition Meets Tradition: A National Mapping of the Physical Education Subject in Primary and Lower Secondary School (5th–10th Grade)]. Elverum: Høgskolen i Innlandet (2018). Report 1:2018.

[33] Standal ØF Moen KM Westlie K. «Ei mil vid og ein tomme djup»?—ei undersøking av innhald og undervisning i kroppsøving på ungdomstrinnet i noreg [«one mile wide and one inch deep»?—An investigation of content and teaching in lower secondary school physical education in Norway]. J Res Arts Sports Educ. (2020) 4(1):34–51. 10.23865/jased.v4.1749

[34] Sticca F Goetz T Bieg M Hall NC Eberle F Haag L. Examining the accuracy of students’ self-reported academic grades from a correlational and a discrepancy perspective: evidence from a longitudinal study. PLoS One. (2017) 12(11):e0187367. 10.1371/journal.pone.0187367 29112979 PMC5675439

[35] Regulations Pursuant to the Education Act. §3-5 Grades in Subject (2006). Available at: https://lovdata.no/forskrift/2006-06-23-724 (Accessed May 5, 2024).

[36] Bjerkan M Rangul V Skjesol K Ulstad SO. Physical activity and depression/anxiety symptoms in adolescents—the Young-HUNT study. Phys Act Health. (2022) 6(1):73–85. 10.5334/paah.185

[37] Guddal MH Stensland SØ Småstuen MC Johnsen MB Zwart JA Storheim K. Physical activity and sport participation among adolescents: associations with mental health in different age groups. Results from the Young-HUNT study: a cross-sectional survey. BMJ Open. (2019) 9:e028555. 10.1136/bmjopen-2018-028555 31488476 PMC6731817

[38] Rangul V Holmen TL Kurtze N Cuypers K Midthjell K. Reliability and validity of two frequently used self-administered physical activity questionnaires in adolescents. BMC Med Res Methodol. (2008) 8(47):1–10. 10.1186/1471-2288-8-47 18627632 PMC2492874

[39] Norwegian Ministry of Education and Research. Læreplan i valgfaget fysisk aktivitet og helse (FAH01-02) [Curriculum in optional subject physical activity and health]. Available at: https://data.udir.no/kl06/v201906/laereplaner-lk20/FAH01-02.pdf?lang=nob (Accessed May 5, 2024).

[40] Little RJA. A test of missing completely at random for multivariate data with missing values. J Am Stat Assoc. (1988) 83(404):1198–202. 10.1080/01621459.1988.10478722

[41] Schafer JL Graham JW. Missing data: our view of the state of the art. Psychol Methods. (2002) 7(2):147–77. 10.1037/1082-989X.7.2.147 12090408

[42] Mehmetoglu M Jakobsen TG. Applied Statistics Using Stata: A Guide for the Social Sciences. London: SAGE (2017). p. 376.

[43] Mehmetoglu M. medsem: a Stata package for statistical mediation analysis. Int J Comput Econ Econom. (2018) 8(1):63–78. 10.1504/IJCEE.2018.10007883

[44] Iacobucci D Saldanha N Deng X. A meditation on mediation: evidence that structural equations models perform better than regressions. J Consum Psychol. (2007) 17(2):39–153. 10.1016/S1057-7408(07)70020-7

[45] Statistics Norway. Marks and National Tests, Lower Secondary School 2021. (2021). Available at: https://www.ssb.no/en/statbank/table/07496/tableViewLayout1/ (Accessed May 5, 2024)

[46] Tinning R. Pedagogy and Human Movement: Theory, Practice, Research. London: Routledge (2010). p. 247.

[47] Hay P Penney D. Assessment in Physical Education: A Sociocultural Perspective. New York: Routledge (2013). p. 160.

[48] López-Pastor VM Kirk D Lorente-Catalán E MacPhail A Macdonald D. Alternative assessment in physical education: a review of international literature. Sport Educ Soc. (2013) 18(1):57–76. 10.1080/13573322.2012.713860

[49] Marmeleira J Folgado H Guardado IM Batalha N. Grading in Portuguese secondary school physical education: assessment parameters, gender differences and associations with academic achievement. Phys Educ Sport Pedagogy. (2019) 25(2):119–36. 10.1080/17408989.2019.1692807

[50] Lyngstad I Bjerke Ø Bang KM Lagestad P. Norwegian upper secondary students’ experiences of their teachers’ assessment of and for learning in physical education: examining how assessment is interpreted by students of different physical abilities. Sport Educ Soc. (2022) 27(3):320–31. 10.1080/13573322.2020.1842728

